# *Aedes* cadherin receptor that mediates *Bacillus thuringiensis* Cry11A toxicity is essential for mosquito development

**DOI:** 10.1371/journal.pntd.0007948

**Published:** 2020-02-03

**Authors:** Jianwu Chen, Karly G. Aimanova, Sarjeet S. Gill

**Affiliations:** Department of Molecular, Cell and Systems Biology, University of California, Riverside, California, United States of America; Universita degli Studi di Pavia, ITALY

## Abstract

*Aedes* cadherin (AaeCad, AAEL024535) has been characterized as a receptor for *Bacillus thuringiensis* subsp. *israelensis* (Bti) Cry11A toxins. However, its role in development is still unknown. In this study, we modified the cadherin gene using ZFN and TALEN. Even though we obtained heterozygous deletions, no homozygous mutants were viable. Because ZFN and TALEN have lower off-targets than CRISPR/Cas9, we conclude the cadherin gene is essential for *Aedes* development. In contrast, in lepidopteran insects loss of a homologous cadherin does not appear to be lethal, since homozygous mutants are viable. To analyze the role of AaeCad in vivo, we tagged this protein with EGFP using CRISPR-Cas9-mediated homologous recombination and obtained a homozygous AaeCad-EGFP line. Addition of *Aedes* Rad51 mRNA enhanced the rate of recombination. We then examined AaeCad protein expression in most tissues and protein dynamics during mosquito development. We observe that AaeCad is expressed in larval and adult midgut-specific manner and its expression pattern changed during the mosquito development. Confocal images showed AaeCad has high expression in larval caecae and posterior midgut, and also in adult midgut. Expression of AaeCad is observed primarily in the apical membranes of epithelial cells, and not in cell-cell junctions. The expression pattern observed suggests AaeCad does not appear to play a role in these junctions. However, we cannot exclude its role beyond cell-cell adhesion in the midgut. We also observed that Cry11A bound to the apical side of larval gastric caecae and posterior midgut cells exactly where AaeCad-EGFP was expressed. Their co-localization suggests that AaeCad is indeed a receptor for the Cry11A toxin. Using this mosquito line we also observed that low doses of Cry11A toxin caused the cells to slough off membranes, which likely represents a defense mechanism, to limit cell damage from Cry11A toxin pores formed in the cell membrane.

## Author summary

A number of receptors for Bt Cry toxins, have been identified and characterized, including cadherin proteins. However, the role of these proteins in the insect is unknown and there have been few efforts to elucidate their function. First, in this study we show that in the mosquito, *Aedes aegypti*, the cadherin protein is essential for development. Secondly, we provide evidence that AaeCad plays a role in the apical membrane and the maintenance of midgut integrity by gene tagging using CRISPR/Cas9, which overcomes the limitation of receptor localization using antibodies in previous studies. These investigations are helpful to further investigate the physiological function of AaeCad. Moreover, this study demonstrated successful tagging of an essential gene with fluorescence protein in a non-model insect. In addition, this study showed that epithelium thinning is possibly a conserved mechanism for host defense against pore-forming toxins, like Cry11A.

## Introduction

*Aedes aegypti* is an important vector of a number of human diseases, including dengue, yellow fever, Chikungunya and Zika [1]. Presently the primary means of controlling mosquito vectors is through use of synthetic chemical insecticides, but increased incidence of insecticide resistance in the field affects their efficacy. Consequently, alternatives such as *Bacillus thuringiensis* subsp. *israelensis* (Bti) are frequently suggested for the control of this insect vector [2,3]. Bti is used worldwide, being the only larvicide certified for mosquito control in the entire Europe. It has also been used for decades in the North American, for example, California and Florida, and is increasingly used in Asia and Africa. It has also been successfully used by the World Health Organization for control of *Simulum*, the vector for onchocerciasis in the Niger delta [4].

Bti is a Gram positive and spore forming bacteria. During its sporulation phase, *Bti* produces three major insecticidal three-domain Cry proteins (Cry4Aa, Cry4Ba and Cry11Aa) and one major cytolytic protein (Cyt1Aa) [5]. Among these, Cry11Aa is one of the most active toxins against *Ae*. *aegypti*. The activity of Cry toxins in lepidopteran and dipteran insects is mediated by different protein receptors. In the case of lepidopteran-specific Cry1A toxins, four different protein receptors have been revealed so far: cadherin [6–8], a glycosylphosphatidyl-inositol (GPI)-anchored aminopeptidase N (APN) [9,10], a GPI-anchored alkaline phosphatase (ALP) [11–14] and more recently ABC transporters [15–17].

Among these receptors, the cadherin, a transmembrane protein, has been the most studied. Binding of a Cry toxin to the toxin binding region (TBR) on a cadherin receptor is required for further toxin cleavage, oligomerization and eventual pore-forming that is critical for intoxication [18]. Presently a number of cadherin mutations have been identified and some confer resistance to the Cry toxins in *Heliothis virescens*, *Pectinophora gossypiella* and *Helicoverpa armigera* [6,19,20]. Moreover, homozygous knockout of the cadherin gene in *H*. *armigera* confers resistance to the Cry1Ac toxin [21]. Many of these mutations in lepidopterans are null alleles, but these insects survive, suggesting the cadherin gene is likely not essential in these insects [22–26]. Based on early reports of these mutants [6,23], we reasoned that knockouts of the *AaeCad* gene would facilitate investigations of its role in Cry11Aa toxicity and larval midgut physiology.

With the exception of ABCC transporters, similar proteins from dipteran insects have been identified as receptors for mosquitocidal Bt toxins [27]. In fact, for the Cry11A toxin a cadherin (AaeCad, AAEL024535), APNs and ALPs have been identified as receptors, and all three proteins are involved in the mechanism of Bti toxicity to *Ae*. *aegypti* larvae. Cells expressing the AaeCad protein show increased sensitivity to Cry11A toxin, and transgenic mosquitoes with silenced AaeCad expression are more tolerant to Cry11A toxin, but not to the Cry4B toxin [28,29]. Although cadherins play an essential role in the toxicity of Cry toxins, their physiological function(s) are unknown in any insect.

Recent genome editing tools provide new opportunities to investigate gene function in non-model insects. The gene editing tools, sequence-specific zinc finger nucleases (ZFN) [30] and transcription activator-like effectors nuclease (TALENs) [31], provided us with the opportunity to elucidate the role of AaeCad. Using both ZFN and TALEN we could successfully obtain deletions in the *AaeCad* gene. However, while we could successfully obtain heterozygous mutants, we could not obtain homozygous mutants.

We therefore hypothesized that AaeCad plays a role in cell-cell adhesion thus being essential for mosquito development. To test this hypothesis we generated C-terminal EGFP-tagged AaeCad homozygous mutants. To do so, we used the clustered regularly interspaced palindromic repeats (CRISPR) associated systems [32]. Unlike ZFN and TALEN, CRISPR/Cas9 functions as a monomer and is more susceptible to off-target issue, but is more amenable to homologous recombination and hence gene tagging [33].

Using CRISPR-Cas9 we generated EGFP-tagged AaeCad homozygous mutants. We then monitored the expression of AaeCad-EGFP during *Aedes* mosquito development in these mutants. We also analyzed its tissue and subcellular localization and response to Cry11Aa intoxication. Our studies suggest that AaeCad plays a fundamental role in the mosquito development but is not involved in septate junctions.

## Methods

### Ethics statement

Mice were used to feed mosquitoes. The protocol for this was approved by the IACUC of University of California, Riverside (UCR).

### Purification and activation of Cry11Aa toxin

Cry11Aa toxin inclusions were isolated from a recombinant strain that was transformed with pCG6 [34]. Briefly, this *B*. *thuringiensis* strain was grown in nutrient broth sporulation medium containing 12.5 μg/ml erythromycin at 30°C. Following cell autolysis, the spores and inclusions were harvested, washed three times with 1 M NaCl plus 10 mM EDTA, pH 8.0 and centrifuged. The resulting pellet was resuspended in 30 ml of the same buffer and purified by NaBr gradients as previously described [35]. The purified inclusions were solubilized in 50 mM Na_2_CO_3_, pH 10.5. Then the solubilized toxins were activated by trypsin (1:20, w/w). The activated Cry11Aa toxin was then purified by ion-exchange chromatography (Mono Q, FPLC).

### ZFN design and synthesis

**Fig 1:**
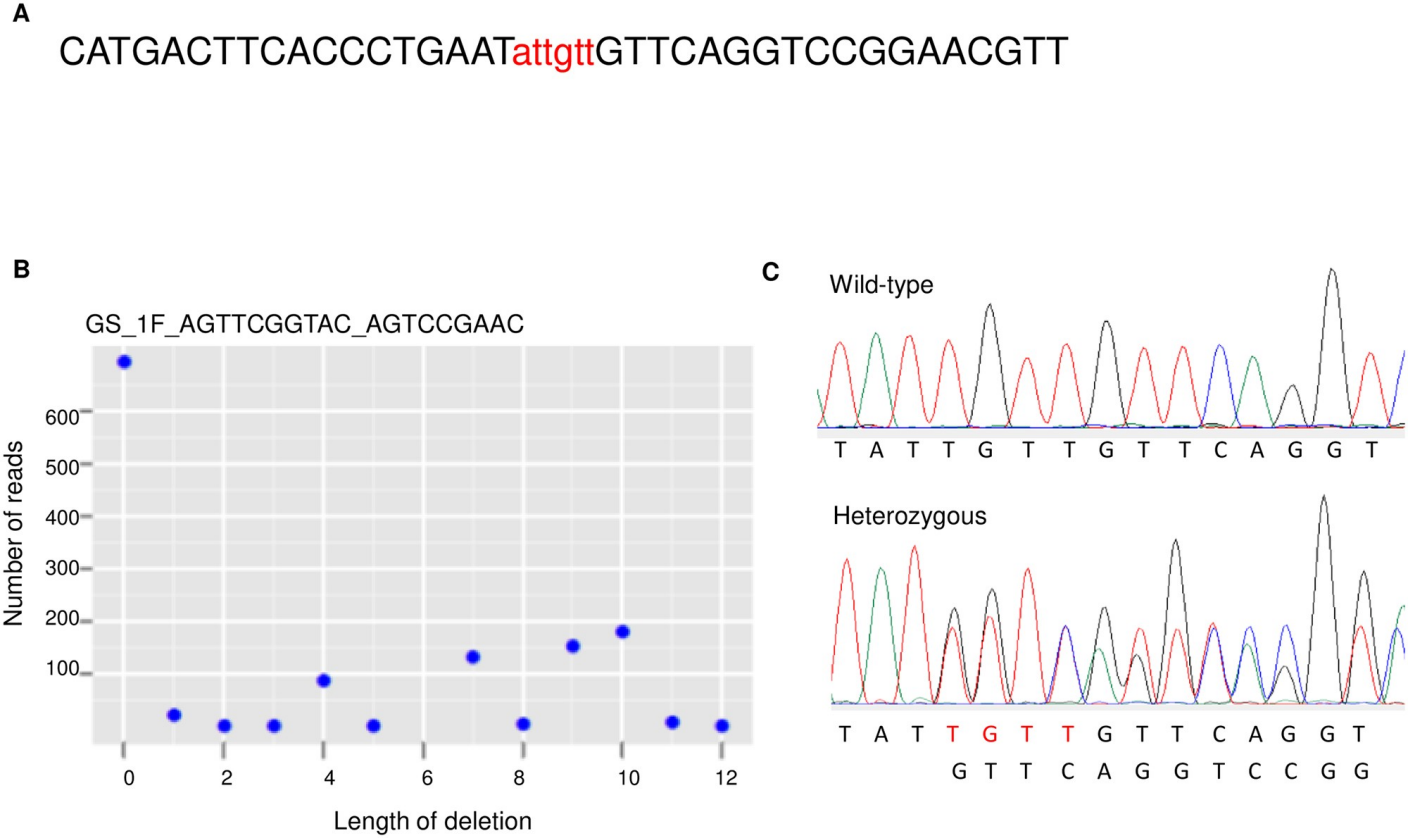
Use of ZFN results in efficient editing of the *AaeCad* gene. *A*. A ZFN construct with zinc finger nuclease binding site (in black, uppercase) and target site (in red, lower case). ***B***. After G1 mosquitoes were genotyped and the generated data was analyzed and plotted. Profiling of the deletion length and the mutation rate is shown for a representative group of G1 mosquitoes. ***C***. A chromatogram of a ZFN-targeted *AaeCad* fragment from wild-type Orlando strain mosquitoes and heterozygous mutant with 4nt-deletion. For ZFN-targeted *AaeCad* fragment from the heterozygous mutant, the overlapping chromatography peaks are displayed after the 4nt deletion.

To knock out the *AaeCad* gene in *Aedes* mosquitoes, we amplified the target DNA fragment from wild-type Orlando mosquitoes by PCR. After the sequences were confirmed by PCR product sequencing, we designed two ZFN constructs (Fig 1A): CATGACTTCACCCTGAATattgttGTTCAGGTCCGGAACGTT (Exon 4) (ZFN binding sites/cut sites/binding sites); TGCTCCCATTTGCTATGAtttgaaAACGGAAGTGGCTGCC (ZFN binding sites/cut sites/binding sites). We had two ZFN constructs made and tested by Sigma-Aldrich (St. Louis, MO, USA). The mRNA was made by Sigma, and stored at -80°C until used for embryo injections.

### TALEN plasmid preparation and mRNA synthesis

**Fig 2:**
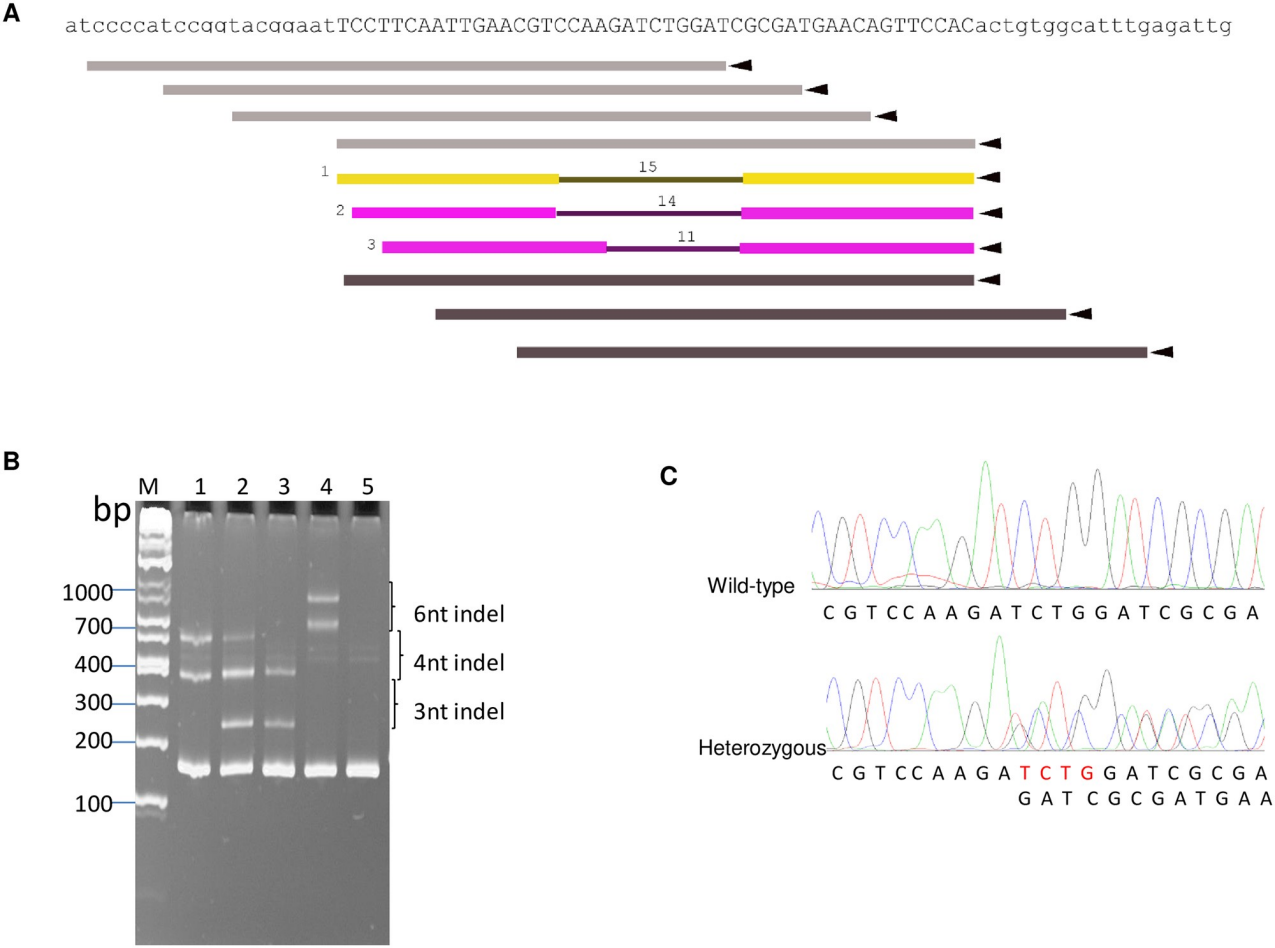
TALEN mediated knockout of the *AaeCad* gene. ***A***. In this TALEN design schematics shows *AaeCad* gene sequence targeted by TALEN. ***B***. G2 mosquitoes were genotyped by native heteroduplex mobility assay (HMA). Four out of 117 mosquito groups analyzed had 3nt, 4nt, 6nt or 3nt & 4nt deletions, respectively. Lane M, DNA marker; Lane 1, sample mosquito #31M with 4nt indel; Lane 2, sample #32M with 3nt and 4nt indel; Lane 3, sample #41 with 3nt indel; Lane 4, sample #70F with 6nt indel; Lane 5, sample #24F with no indel. ***C***. PCR product sequencing from a heterozygous mutant displayed overlapping chromatogram peaks, but those from wild-type Orlando strain mosquito showed single chromatogram peaks.

As an alternative we also attempted to knock out the *AaeCad* gene by TALEN. In this case we truncated *AaeCad* to delete the toxin binding regions and the intracellular domain. DNA fragments between cadherin repeats 6 and 7-coding sequences (in Exon 10) was amplified from *Aedes* Orlando strain mosquito and PCR product was sequenced and aligned with the *AaeCad* sequence from the Liverpool strain. Then the left (TCCTTCAATTGAACGT) and right arms (CGATGAACAGTTCCAC) targeting this fragment with a 15 nt spacer were designed by the TALENhit program (Fig 2A). The TALEN construct with corresponding left and right arms was synthesized by Cellectis Biosearch (Paris, France), and the TALEN cleavage activity was validated before use. After the TALEN plasmid was linearized by *Hin*dIII, the capped TALEN left and right arm 2.6 kb mRNA was synthesized by mMESSAGE mMACHINE T7 Ultra Kit (Thermo Fisher Scientific, MA, USA). These mRNA were further processed by polyA tailing. The resultant mRNA was purified by MEGAclear Kit (Thermo Fisher Scientific, MA, USA), and the purified mRNA was then used for mosquito embryo injections.

### Heteroduplex mobility assay (HMA)

To verify the presence of indels in the *AaeCad* gene caused by TALEN injection, a heteroduplex mobility assay [36] was performed. The *AaeCad* gene-specific forward primer (5’-GACTCAACACTCCCTGCAGTAG-3’) and reverse primer (5’-GAATTTCTGCAAGTCCGGAAACG-3’) were used for amplification of partial *AaeCad* fragment with the TALEN target sites. Then the PCR amplicons were analyzed in 15% poly-acrylamide gel. Based on the denaturing and annealing, PCR product with strands, which are not fully complementary would form the heteroduplex, but those with fully complementary stands formed the homoduplex. Because of an opened single strand configuration surrounding the mismatch region, the heteroduplex migrated more slowly and was separated from the homoduplex in the gel. Also the similar heteroduplex with a larger deletion in one strand would move slower in the gel.

### sgRNA design, synthesis and *in vitro* cleavage assay

**Fig 3:**
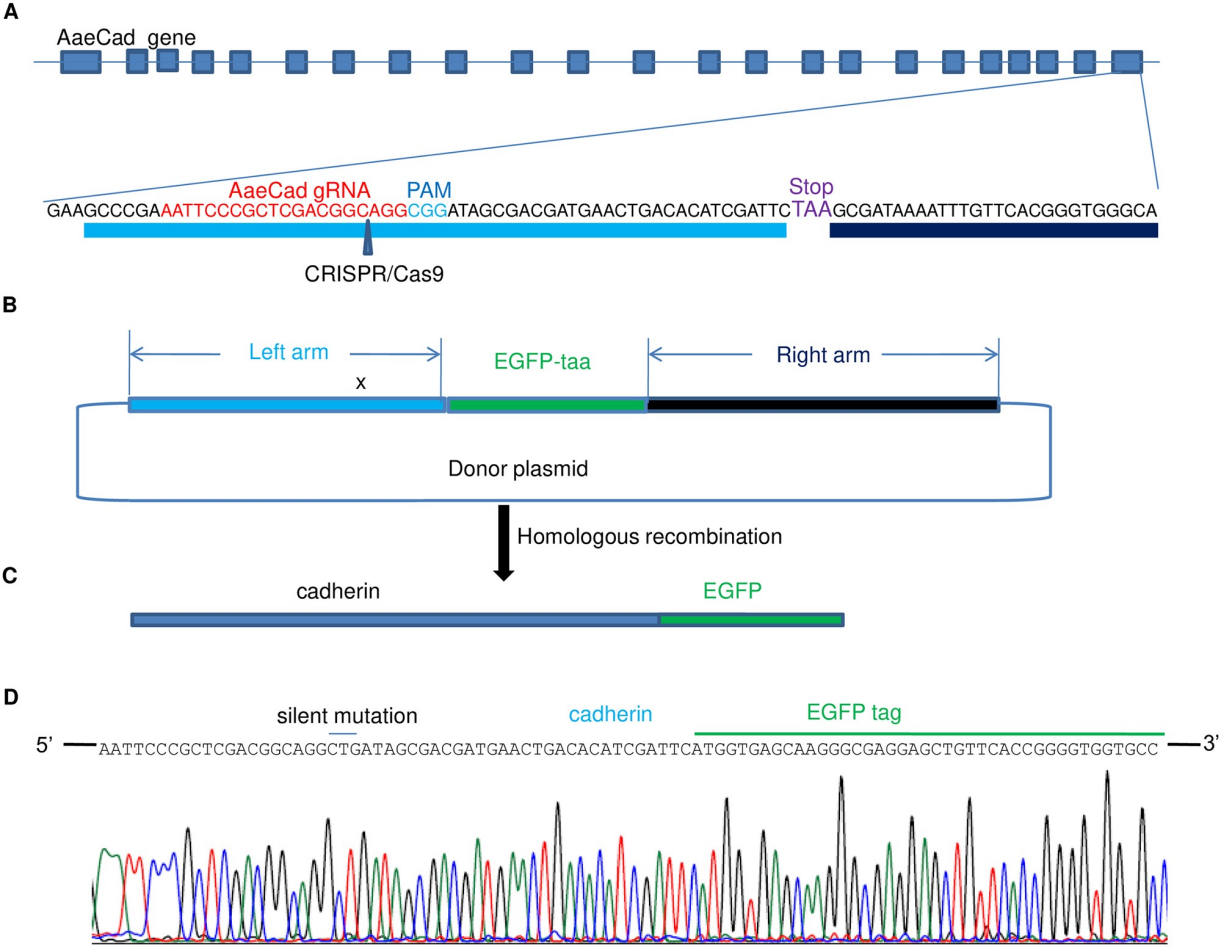
CRISPR-Cas9 mediated EGFP tagging at the 3’ end of the last coding exon of *AaeCad* gene results in an in frame fusion. ***A***. sgRNA (in red) and PAM sequences (in blue) were designed before the stop codon (in purple) on the last exon of *AaeCad* gene. ***B***. This donor plasmid for homologous recombination (HR) contains 1000bp of left arm, 720 bp of EGFP ORF and 1000bp of right arm. To prevent Cas9 cleavage on the left arm in the plasmid, a silent mutation (CGG → CTG) was introduced into the PAM sequence on the left arm. ***C***. When the homologous recombination occurred, the EGFP was introduced immediately after the *AaeCad* gene with the aid of the left and right arms. ***D***. The sequencing results of gDNA showed the *AaeCad* gene had been successfully tagged with EGFP and no mutation was introduced into the *AaeCad* gene except for the silent mutation that was intentionally introduced.

A sgRNA target sequence was designed using the CHOPCHOP program (http://chopchop.cbu.uib.no/) based on 50 bp sequences flanking the stop codon on the last exon of the *AaeCad* gene (Fig 3A). A gRNA most proximate to the stop codon with minimum off-targets and relatively high predicted efficiency was chosen. To generate the sgRNA template, PCR by Platinum High-fidelity Taq polymerase (Thermo Fisher Scientific, MA, USA) was performed with two PAGE-purified oligos, CRISPR_F with the T7 promoter and *AaeCad* target sequence (5’-GAAATTAATACGACTCACTATAGGAATTCCCGCTCGACGGCAGGGTTTTAGAGCTAGAAATAGC–3’) and CRISPR_R with the remaining sgRNA sequences (5’-AAAAGCACCGACTCGGTGCCACTTTTTCAAGTTGATAACGGACTAGCCTTATTTTAACTTGCTATTTCTAGCTCTAAAAC–3’). After agarose gel electrophoresis, the PCR product was purified by Zymoclean Gel DNA Recovery Kit (Zymoresearch, Irvine, CA, USA). *In vitro* transcription of purified PCR product was done using a MEGAscript Transcription Kit (Ambion, Foster City, CA, USA). The resultant mRNA was purified by a MEGAclear Kit (Ambion, Foster City, CA, USA). The synthesized sgRNA samples were immediately aliquoted and stored at -80°C before use.

To test the activity of synthesized sgRNA, an *in vitro* cleavage assay was performed. The synthesized sgRNA, 150ng, 100ng of a plasmid pActin-Aaecad DNA [28] and 300 ng of Cas9 protein (PNA Bio Inc., Thousand Oaks, CA, USA) were incubated at 37°C for 1 hr in 10 μL of 1x NEB buffer 3.1 (New England Biolabs, Ipswich, MA, USA). Plasmid DNA in the absence of Cas9 protein and sgRNA or with the presence of either Cas9 or sgRNA was used as control groups. All samples were treated with 4μg of RNase at 37°C for 15 min before running DNA gel analysis. Then 1μl of stop solution (30% glycerol, 1.2%SDS, 250mM EDTA, pH 8.0) was added into the reaction mixture and incubated at 37°C for another 15 min. Then all the samples were analyzed on 1% agarose gel.

### High resolution melt (HRM) analysis

To assess sgRNA efficiency *in vivo*, 100 embryos were injected with a Cas9-gRNA mixture. Of these 24 hatched and their genomic DNA and that of three wild-type Orlando larvae were individually extracted by DNeasy Tissue and Blood Kit (Qiagen, Hilden, Germany). The concentration and purity of genomic DNA was measured in a Nanodrop 2000 spectrophotometry (Thermo Fisher Scientific, Waltham, MA, USA). High resolution melt analyses were performed by Precision Melt Supermix (Bio-Rad, Hercules, CA, USA) using optimized forward (GTACTAACTCAACTTTATGGC) and reverse (GGCATTTTCCCCCCAACCTG) primers. In 10μL reaction was included with 1 μL of primer (2 μM), 4 μL of genomic DNA (50 ng) and 5 μL of precision melt supermix. Real time PCR was run in Bio-Rad’s CFX 96 under the following conditions: one cycle of 95 C, 2 min; 40 cycles of 95 C, 10 sec, 60 C, 30 sec and 72 C, 30 sec; 1 cycle of 95 C, 30 sec, 60 C, 1 min and 65–95 C in 0.2 C increments, 10 sec/step. The generated data files were imported into Precision Melt Analysis software for HRM analysis based on the thermal denaturation properties of double-stranded DNA. The normalized melt curves were grouped with the wild-type samples as references.

### Homologous recombination (HR) plasmid design and synthesis

After the sgRNA efficiency was confirmed *in vitro* and *in vivo*, the flanking 1500 bp fragments were amplified by PCR and sequenced. The plasmid used for HR was designed based on these sequences. It consists of a 1000 bp left arm immediately before the stop codon, a 1000bp right arm right after the stop codon and a 720 bp EGFP ORF in between the left and right arms (Fig 3B). To avoid cleavage of the plasmid by the sgRNA, a silent mutation on the PAM sequence in the left arm was introduced into the plasmid (CGG → CTG). This construct was synthesized and cloned into pUC57 plasmid by GenScript (Nanjing, China). Plasmid DNA was prepared by EndoFree Plasmid Maxi Kit (Qiagen, Hilden, Germany) and kept for future use in -20°C.

### Analyses of *Aedes* mosquitoes in *AaeCad* knockouts

Three days after ZFN or TALEN mRNA were injected into *Ae*. *aegypti* embryos by the Insect Transformation Facility at University of Maryland (Rockville, MD, USA), G0 eggs were hatched and reared with a mixture of dog food and yeast (3:1) in deoxygenated tap water at 29 °C, 16:8h light: dark, and 50% humidity. When they became adults, 3 females were mated with 3 males in a group (S1A Fig). All these females were separated for egg collection. Once G1 larval mosquitoes turned to adults, they were in-cross mated. After their eggs were collected three times, half of the eggs were hatched and the resultant G1 adult mosquitoes were collected for genomic DNA extraction. A PCR product library was made by mixing all the PCR products from separate groups and sent for PCR product profiling in Beijing Genomics Institute (Beijing, China). The generated genotyping data was analyzed by the Bioinformatics facility at University of California, Riverside (UCR). Then a group with relatively high mutation rate was chosen and another half of their eggs were hatched. When G2 mosquitoes became adults, their fresh pupal casings were collected for genomic DNA extraction. After determining the purity and concentration, the DNA was used as the template for nested PCR by Choice Taq Blue Mastermix (Deville Scientific Inc., Metuchen, NJ, USA) under the following conditions: one cycle of 94 C, 3 min; 35 cycles of 94 C, 45 sec, 60 C, 30 sec and 72 C, 1 min; 1 cycle of 72 C, 10 min. Then the final PCR product was sent for sequencing at the Institute of Integrative Genome Biology (IIGB) at UCR. G2 mosquitoes with the desired 4nt mutation were mated with the wild-type Orlando mosquitoes for propagation and their eggs again were collected. For G3 mosquitoes, a heterozygous mutant was mated with another heterozygous mutant of the opposite sex expecting 25% of the progeny would be homozygous mutants in G4 mosquitoes. Although this process was repeated several times, no homozygous mutants were obtained.

To C-terminally tag the *AaeCad* with EGFP two embryo injections were performed by the Insect Transformation Facility at University of Maryland; the first with a mixture of gRNA, Cas9 protein and HR donor plasmid, and the second had in addition Rad51c mRNA (Table 2). For both injections, two G0 male adult mosquitoes were mated with 2 female G0 adult mosquitoes. After egg laying all male and female mosquitoes were collected and their gDNA analyzed for EGFP knockin using nested PCR. Eggs from a positive G0 group were hatched and 2 G1 females were mated with 2 G1 males. After their eggs were collected, the gDNA of all adult mosquitoes was again analyzed by PCR for the EGFP knockin. Eggs from one positive G1 group were hatched and single pair-mating was done for the G2 adult mosquitoes. Again, after G3 eggs were collected, G2 adult mosquitoes were genotyped by PCR. Then G3 eggs from one group with both positive male and female were hatched and G3 adult mosquitoes mated. After G4 eggs were collected, all eggs from the same female were hatched together and screened for a homozygous group by examining for green fluorescence in the larval midguts using fluorescence microscopy (Nikon SMZ1500). After screening for a few possible homozygous groups, G5 adult mosquitoes were sib mated for propagation to obtain a stable mosquito line. This homozygous line was used for further investigations.

### Immunohistochemistry

*Ae*. *aegypti* larval guts were dissected in phosphate buffered saline (PBS), transferred into 4% paraformaldehyde (PFA) and fixed at 4°C overnight. The tissues were then washed in PBST (PBS plus 0.1% Triton X-100) three times for 30 min, incubated in 15% sucrose solution (15% sucrose in PBS) overnight followed by 30% sucrose solution overnight. The tissues were embedded first in OTC compound (Sakura Finetek USA Inc., Radnor, PA, USA) on dry ice, and then the embedded sections were frozen in -80°C. Sections, 10 μm thick, were made using a Cryostat Leica CM950 (Wetzlar, Germany), placed on poly-L-lysine (Sigma-Aldrich, St. Louis, MO, USA) slides coated with 1% gelatin (Becton Dickson, Franklin Lakes, NJ, USA), and then the sections were dried at 40°C for 1 h before processing.

For immunolocalization, the sections were pre-wetted in PBST for 10min followed by incubation in blocking buffer (2% goat serum in PBST) at room temperature for 1hr. The sections were incubated with primary antibodies at 4°C overnight and then washed as described above. The primary antibodies used here are rabbit polyclonal antibodies to AaeCad (1:100) prepared in our lab, but mouse monoclonal antibodies to Armadillo (N27A1) were obtained from the Developmental Studies Hybridoma Bank (DSHB) (Iowa city, Iowa, USA) and were used for staining at a final concentration of 5μg/mL. All sections were incubated in the dark with either Alexa Fluor 647 goat-anti-rabbit IgG or Alexa Fluor 555 goat-anti-mouse IgG secondary antibodies (Thermo Fisher Scientific, MA, USA) and DAPI at 0.1μg/mL at room temperature for 1 h. After washing three times in the same buffer, the sections were mounted in Aqua-Poly/Mount medium (Polysciences, Inc., Warrington, PA, USA). However, for the toxin binding assay, Cry11A (10ng/μL) was incubated with all sections followed by Cry11A toxin detection using anti-Cry11A antibody and an Alexa Fluor 647 goat-anti-rabbit IgG. Images were obtained using a SP5 Inverted confocal microscope (Leica, Wetzlar, Germany) in IIGB at UCR. All images were imported into Adobe Photoshop for brightness adjustment and proper annotation.

### Bioassays

Bioassays were performed with fourth instar of *Ae*. *aegypti* larvae using a spores/inclusions suspension. Briefly, 20 early 4^th^ instar larvae were placed in water in 10-oz plastic cups (Costar, USA) to which Cry11Aa suspensions (5.6 ± 0.52 x 10^8^ CFU/mL) of 20, 40, 80, 160 and 320 μL were added in a total volume of 200 ml, respectively. Mortality was recorded after 24-h incubation at room temperature. All bioassays were performed at least three times. Dose-response values were analyzed by a probit program (EPA) and plotted using Origin (Origin Lab, Northampton, MA).

## Results

### The cadherin gene is essential for *Aedes* development

To knock out the cadherin gene using ZFN, we designed and synthesized a ZFN construct (Fig 1A). Since construct 1 had higher ZFN activity, mRNA synthesized from this construct was used for embryo injections. Of 741 embryos injected with mRNA only 59 hatched, with a hatch rate of about 7.9%, (Table 1). Eventually, only 54 G0 developed into adult mosquitoes. These mosquitoes were mated and eggs collected as described (S1A Fig). Half of the G1 eggs collected were hatched and used for genotyping. The genotyping data (Fig 1B) showed that 43 out of 54 G1 matings carried an indel, indicating a high mutation rate, about 79.6% (Table 1). Then we focused on one group with a relatively high mutation rate and crossed them with wild-type Orlando mosquitoes. We found the G2 mosquitoes had various nucleotides deleted, but only heterozygous mutants with a 4nt deletion between 1067 and 1070nt in ORF (Fig 1C) were first processed. When the heterozygous mutants were crossed with each other, we failed to obtain any homozygous mutants from G4 mosquitoes. Although we analyzed four more generations, we were unable to obtain any homozygous mutants. Further, when we tested other mutants with a 2nt, 8nt or 16nt deletion at G2, we could not obtain any homozygous mutants at G4 either.

**Table 1:** Use of either ZFN or TALEN led to knockout of the *Aedes* cadherin gene, but ZFN is far more efficient.

Group	Components	Injected embryos	G0 larval survivors (%)	G1 Indel rate (%)
#1	Aacad ZFN	741	59 (7.9%)	43/54 (79.6%)
#2	AaeTALEN	793	267 (34%)	4/117 (3.4%)

When TALEN became available, we tried to knock out the *AaeCad* gene by TALEN at a new site, 3’ downstream of the initial ZFN target site. The TALEN left arm and right arm mRNAs were made and processed by polyA tailing (S1B Fig). The purified tailed mRNAs were mixed and used for embryo injections. In total, 793 mosquito embryos were injected and 267 hatched (hatch rate of 34%) (Table 1). After G2 eggs were collected, G1 mosquitoes were genotyped and analyzed as above for ZFN. Bioinformatics analyses showed 4 out of 117 groups carried 3nt, 4nt, 6nt, 3nt & 4nt deletions, respectively (S1 Table) and their mutation rate ranged from 6.07% to 29.6%. These bioinformatics results were then confirmed by heteroduplex mobility assay (HMA). In addition to a 150 bp band amplified from wild-type Orlando strain, the PCR products from the samples with 6nt, 4nt and 3nt indel had two additional bands with sizes of about 900 & 700 bp, 600 & 370 bp and 370 & 250 bp, respectively (Fig 2B). We analyzed further one group, #31M, which had a 4nt indel between 2682 and 2685 nt in the ORF. The gDNA from pupal casings of the G2 mosquitoes as well as that of all G2 individual mosquitoes from this 31M strain were analyzed. Wild-type gDNA have distinct chromatographic peaks, but those with 4nt deletion showed overlapping chromatographic peaks, indicating their heterozygosity (Fig 2C). The heterozygous mosquitos were mated with wild-type Orlando strain mosquito for propagation. After G3 heterozygous mosquitos were identified, single-pair matings were done to obtain homozygous mosquitoes at G4. However, we could not obtain a homozygous mutant even though we tried an additional four generations of selection. We were also unable to obtain a homozygous knockout of the *AaeCad* gene using ZFN. Taken together, because ZFN and TALEN have much less off-target issues than CRISPR-Cas9 [37], the *AaeCad* gene is very likely essential for normal mosquito development.

### *AaeCad* gene was successfully tagged with EGFP using CRISPR-Cas9-mediated homologous recombination

Since the *AaeCad* gene knockout is lethal for *Aedes* mosquito, we surmised that this gene plays an important role in the mosquito midgut. We fluorescently tagged AaeCad to facilitate monitoring its expression in vivo and help investigate its role in mosquito midgut physiology and in the toxicity of Cry11Aa. Using CRISPR-Cas9 homologous recombination (HR) we successfully tagged the AaeCad protein with EGFP at the C-terminus. To do this, we showed using an *in vitro* cleavage assay that in the presence of both Cas9 and gRNA, the AaeCad in the plasmid was cleaved, but not when only Cas9 or gRNA was used (S2B Fig). Then this gRNA and Cas9 were used for an *in vivo* cleavage assay using high-resolution melt (HRM) analysis. Seven out of 24 survivors showed differential HRM curves (S2C Fig), suggesting the gRNA also worked efficiently *in vivo*. Moreover, as a key player of homologous recombination and DNA repair, Rad51 recombinase has been shown to facilitate increased HR efficiency [38–40]. Hence, *Aedes* Rad51 ORF was amplified from *Aedes* mosquito, and three different isoforms of *Aedes* Rad51, Rad51a, b and c, were identified and cloned into the vector, pcDNA3.1 (S3A Fig). Among them, Rad51c is the longest isoform. Thus, it was used for mRNA synthesis and polyA tail processing (S3B Fig). The purified Rad51c mRNA was also used for embryo injections.

Two embryo injections were made to tag *AaeCad* gene with EGFP. The first was performed with a mixture of gRNA, Cas9 protein and HR donor plasmid, while the second included in addition Rad51c mRNA (Table 2). In total, 512 and 516 mosquito embryos were injected for these two groups and the hatch rate was 24.2% and 22.5%, respectively. Of these, 2 out of 123 G0 survivors from the first injection had EGFP tagged to the *AaeCad* gene, with a correct HR rate of 1.6%. Whereas 3 out of 104 survivors from second injection were detected with EGFP-tagged *AaeCad* gene, with a HR rate of 2.9%. This suggests that while the precise knock-in rate for both injections is relatively low, Rad51c mRNA enhances the precise knock-in rate by about 1.8-fold (Table 2).

**Table 2:** CRISPR-Cas9 mediated homologous recombination to generate an EGFP tagged cadherin gene is more efficient with Rad51.

Group	Components *	Injected embryos	G0 larval survivors (%)	G0 HR rate (%)	G1 HR rate (%)
#1	donor plasmid	512	124 (24.2%)	2/123 (1.6%)	N/A
#2	donor plasmid/Rad51 mRNA	516	116 (22.5%)	3/104 (2.9%)	5/65 (7.7%)

* In addition to gRNA and Cas9 protein.

To confirm that the *AaeCad* gene was correctly tagged, PCR products from positive mosquitoes were sequenced and analyzed. Sequencing results confirmed the *AaeCad* gene was successfully tagged in frame with EGFP and a silent-mutation (CGG → CTG) was also introduced into the *AaeCad* gene by HR (Fig 3C and 3D). Eggs from G0 mosquitos were hatched, but eggs from only one out of 5 positive groups successfully hatched and grew into adults. Eggs from the other 4 groups either did not hatch or did not develop into adults. For G1 mosquitoes, only five out of 65 G1 survivors were positive, so the heritable rate is 7.7% (Table 2). However, when G2 progenies derived from this positive G1 female were screened by nested PCR, the results showed that half of the mosquitoes had EGFP knock in. When the native *AaeCad* gene-specific primers were used for PCR, the PCR product could be amplified from all samples, indicating that all these positive mosquitoes were still heterozygous. After single-pair mating and screening through an additional two generations, we succeeded in getting an EGFP-tagged cadherin mutant homozygous line. This homozygous line was then used for further investigations. Our results demonstrated successful tagging of an essential gene in non-model insects.

### AaeCad protein is expressed in a tissue specific manner during mosquito development

In the homozygous EGFP-tagged line, AaeCad protein expression was readily observed in all stages of larval development except in the neonates. In 2^nd^, 3^rd^ and 4^th^ instar larvae, Aae-EGFP was always expressed in posterior midgut, but its expression pattern in the cardia and gastric caecae was variable (S4A, S4B, S4C and S4D Fig). In 2^nd^ instar larvae, AaeCad-EGFP was highly expressed in cardia (S4A Fig). In 3^rd^ instar larvae, some Aae-EGFP was observed in gastric caecae (GC), but most of expression was in the cardia (S4B Fig). But in early 4^th^ instar larvae, AaeCad-EGFP was mostly expressed in gastric caecae (S4C Fig). Interestingly, in late 4^th^ instar larvae, most AaeCad-EGFP was expressed in cardia again (S4D Fig). Thus, it is clear that AaeCad was first expressed in the cardia and then moved to the gastric caecae gradually with the larval development, but AaeCad protein migrated back to cardia before pupating. However, since the cuticle blocks green fluorescence we were unable to monitor in vivo expression during the pupae and adult stages (S4E, S4F, S4G and S4H Fig).

**Fig 4:**
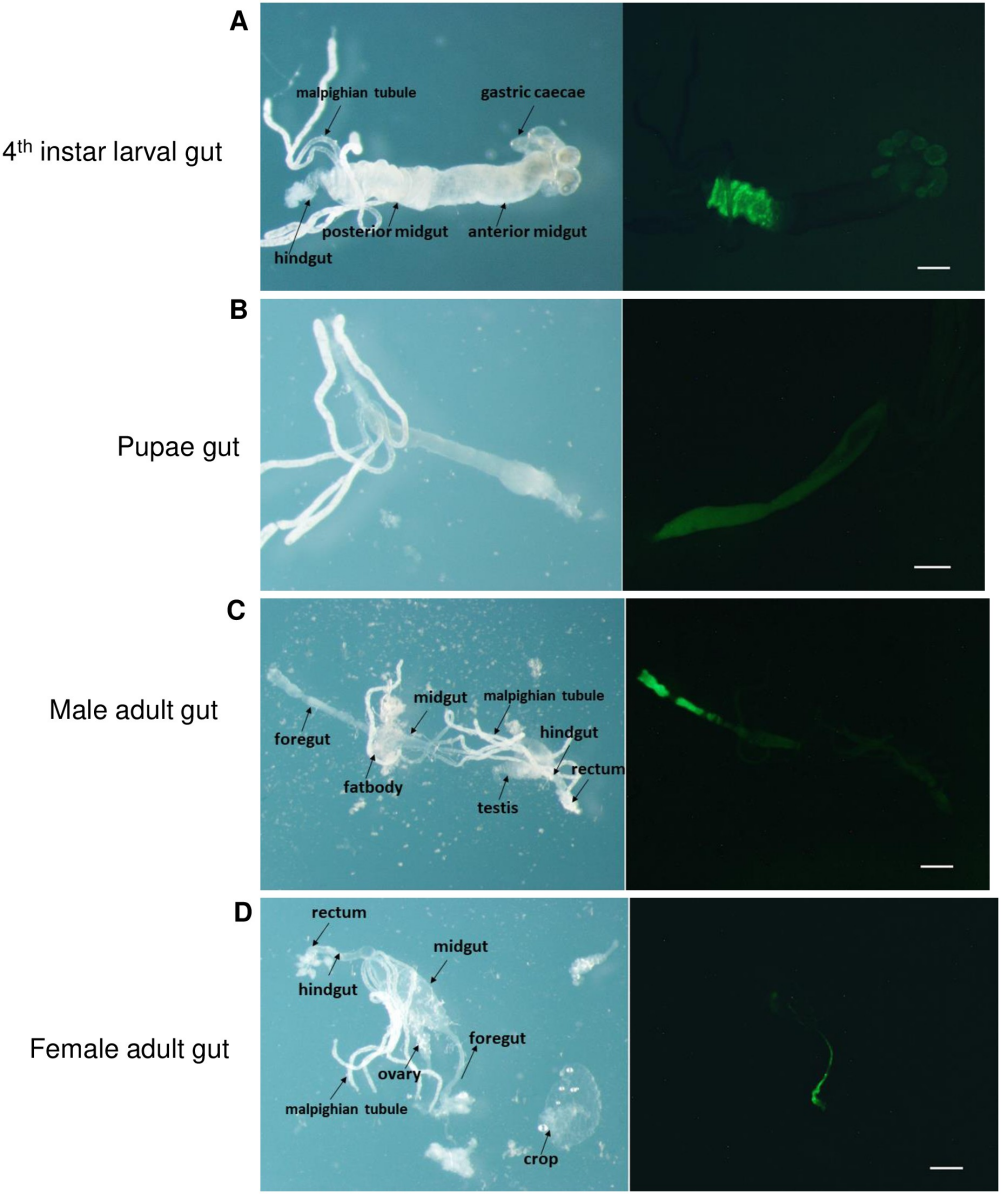
Visualization of AaeCad-EGFP in whole mounts of dissected guts from larva, pupa and adult male and female homozygous *Aedes*. ***A***. AaeCad protein localization in early 4^th^ instar larval gut. Expression is observed primarily in the gastric caecae and the posterior midgut. ***B***. AaeCad protein expression in pupal gut. ***C***. AaeCad protein localization in adult male gut; D. AaeCad protein localization in adult female gut. Expression in both the male and female was observed primarily in the foregut. Since the tissues were in PBS buffer the samples move ever so slightly under the different filters, preventing use of the merge function. Bar: 50μm.

To more clearly observe AaeCad expression patterns, mosquito midguts were dissected. As in whole larvae, dissected midguts from early 4^th^ instar larvae showed strong AaeCad expression in the gastric caecae and in the posterior midguts (Fig 4A). Also, in dissected midguts from both male and female adult mosquitoes showed AaeCad is expressed strongly in the foregut but weakly in the midguts (Fig 4C and 4D). But in the dissected pupal gut, EGFP expression was less intense (Fig 4B). No AaeCad expression was observed in tissues other than guts in larvae, pupae and adults. Tissues analyzed included ovary, testes, the hind gut and Malpighian tubules. Therefore, the AaeCad protein is expressed in a tissue specific manner.

### AaeCad localizes primarily to the apical side of larval gastric caecae and posterior midgut (PM) cells

**Fig 5:**
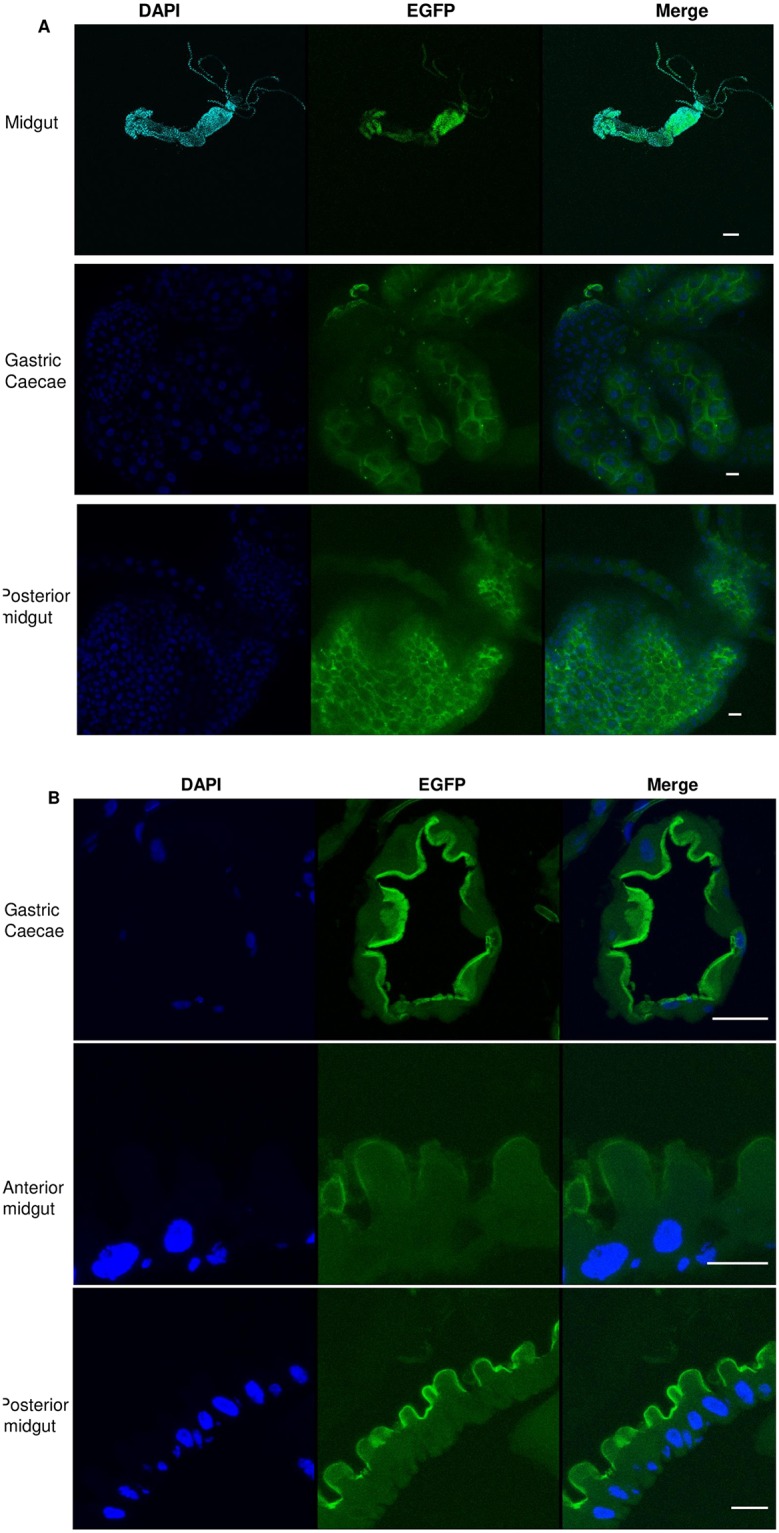
AaeCad protein is localized primarily in gastric caecae and posterior midgut of early 4^th^ instar larvae. ***A***. Whole mount images under low magnification showed that strong AaeCad-EGFP expression in the gastric caecae (GC) and posterior midgut cell membrane. ***B***. Cross section images showed high AaeCad-EGFP expression in the gastric caecae (GC) and posterior midgut and low expression in the anterior midgut. In the gastric caecae and midgut, EGFP-tagged AaeCad is specifically expressed in the epithelial cell membrane. DAPI stains the nucleus. All images were collected using an SP5 Inverted confocal microscope. Bar: 50μm.

To determine if AaeCad plays a role in cell-cell adhesion, we analyzed the subcellular localization of AaeCad in larval guts. Whole larval midguts were imaged using a confocal microscope (Leica SP5). Strong AaeCad-EGFP expression was observed in gastric caecae (GC) and posterior midgut (PM) cell membranes (Fig 5A). Notably, cells in gastric caecae are larger than those in posterior midgut. To further characterize the subcellular localization of AaeCad protein, the larval midguts were embedded and frozen sections from different parts of midgut were analyzed. High AaeCad-EGFP expression was observed on the apical side of gastric caecae and posterior midgut (PM) cells and lower expression in the anterior midgut (AM) (Fig 5B).

**Fig 6:**
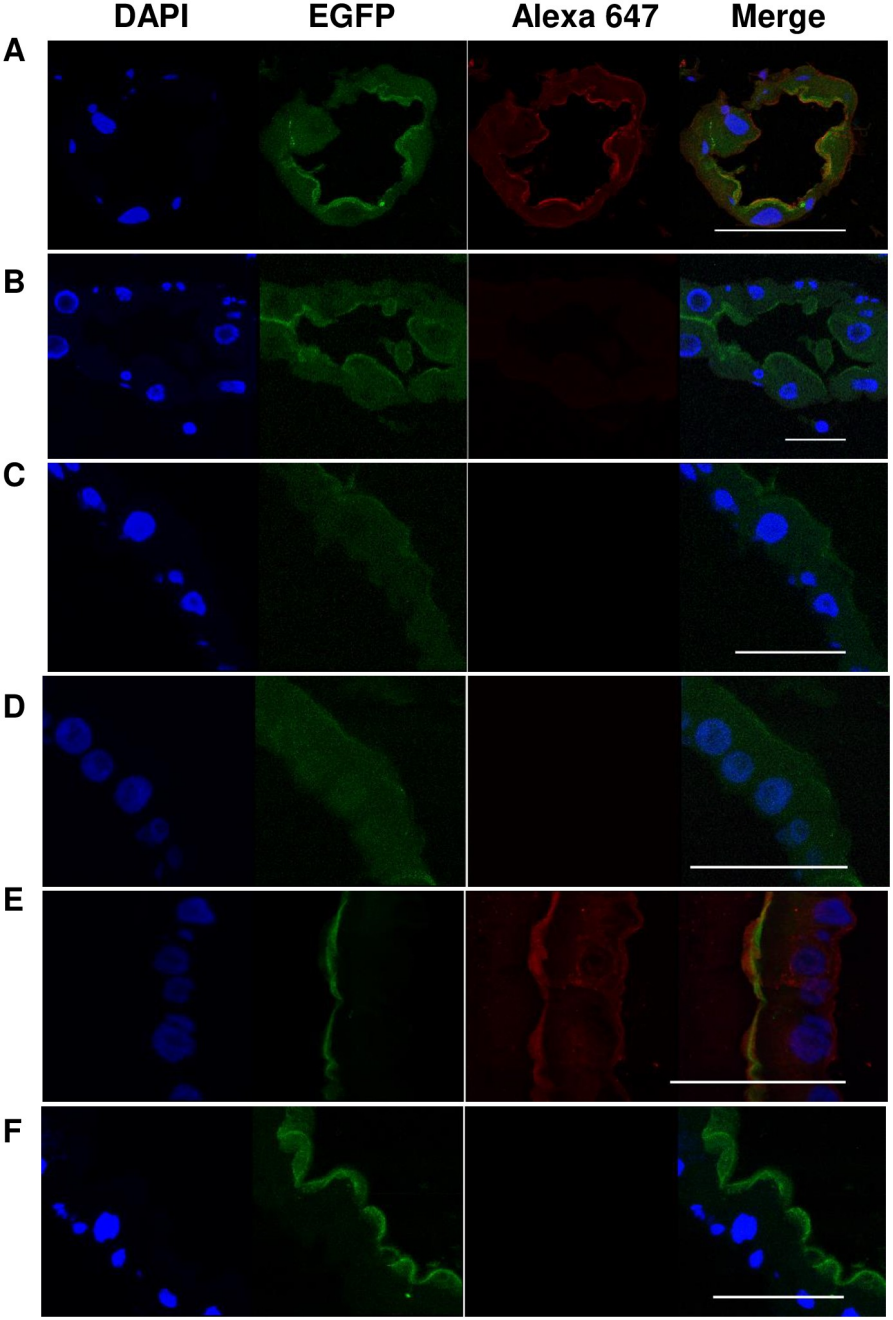
An anti-AaeCad polyclonal detects the EGFP-tagged AaeCad. Anti-AaeCad polyclonal antibody could detect the EGFP-tagged AaeCad in gastric caecae (A) and posterior midgut (E), but not in the anterior midgut (C). Whereas, when only the Alexa 647-labeled antibody was used, no fluorescence was detected in the gastric caecae (B), anterior midgut (D) and posterior midgut (F). Bar: 50μm, but is 25μm in panel E.

We previously showed that an anti-cadherin antibody binds to the epithelial layer of gastric caecae and the posterior gut [41]. To confirm that AaeCad-EGFP indeed is expressed at the same sites as localized by the anti-cadherin antibody (Alexa 647), we analyzed the expression of AaeCad in the EGFP-tagged mosquito line. We observed that in the gastric caecae (Fig 6A) and posterior midguts (Fig 6E), the EGFP-tagged cadherin co-localizes with the antibody-detected cadherin. However, since expression of AaeCad protein in the anterior midgut was low as observed with AaeCad-EGFP (Fig 5B), its expression was not detected with the anti-AaeCad antibody (Fig 6C) and as previously reported [41]. In the absence of anti-AaeCad antibody, no fluorescence was observed in gastric caecae (Fig 6B), anterior midgut (Fig 6D) and posterior midguts (Fig 6F).

### Cry11A toxins caused the loss of midgut membranes

**Fig 7:**
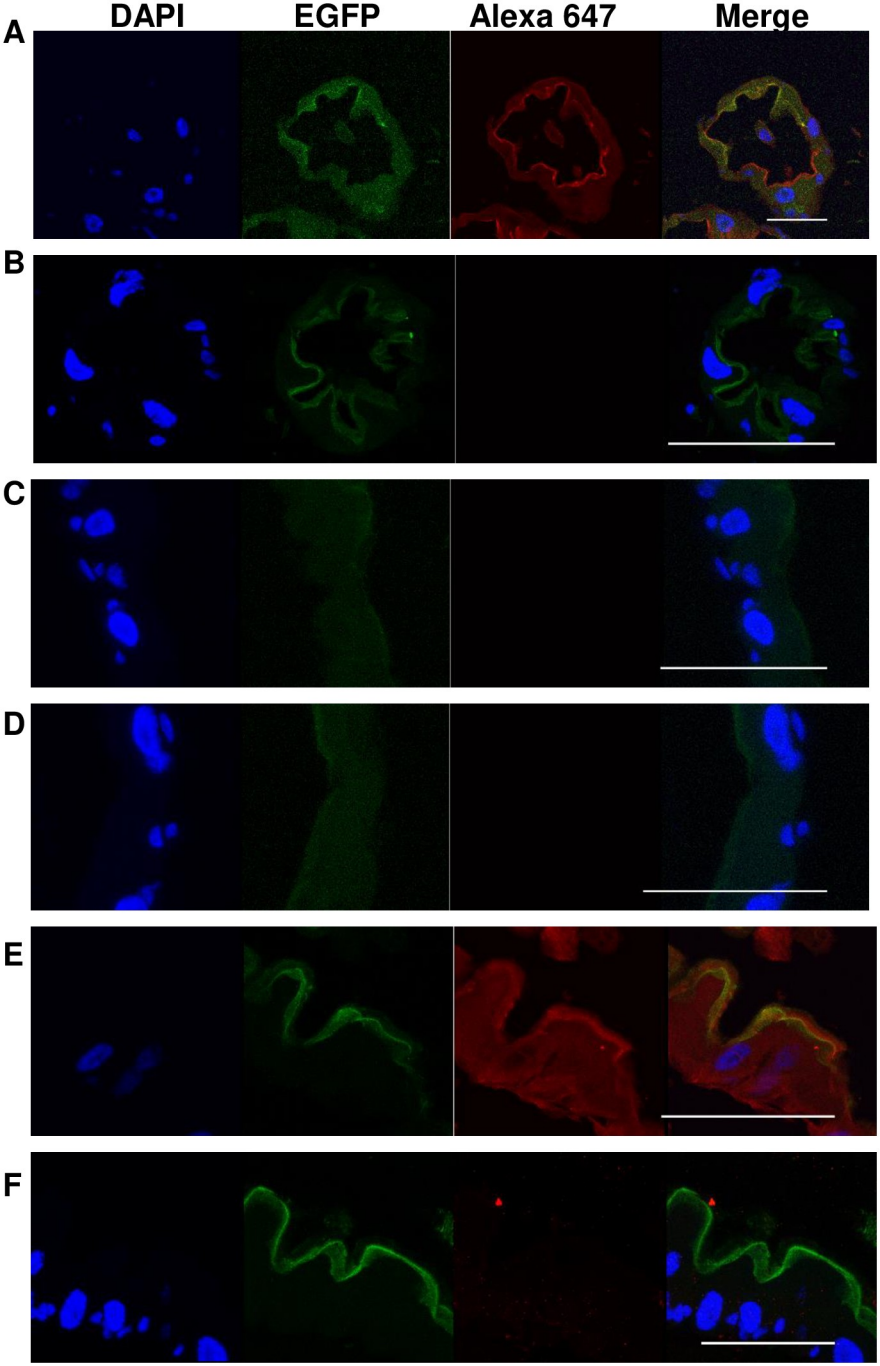
EGFP-tagged AaeCad co-localizes in the larval gut cells with the Cry11Aa toxin. The Cry11A toxin bound to the AaeCad-EGFP in the gastric caecae (A) and posterior midgut (E), but not in the anterior midgut (C). However, when the Cry11A toxin was absent, the fluorescence could not be observed in the gastric caecae (B), anterior midgut (D) and posterior midgut (F). Bar: 50μm, but is 25μm in panels E and F.

We have previously reported that the Cry11Aa toxin binds the epithelial layers of the gastric caeca and the posterior midgut [41]. Here we show that Cry11A toxin binds to the AaeCad-EGFP in the gastric caecae (Fig 7A) and the posterior midguts (Fig 7E), but not in the anterior midgut (Fig 7C). In the absence of Cry11A toxin but in the presence of the anti-Cry11A antibody, no fluorescence was observed in the gastric caecae (Fig 7B), anterior midgut (Fig 7D) and posterior midgut (Fig 7F). Hence, the toxin binding ability of the AaeCad receptor C-terminally tagged with EGFP is unaltered. Further, bioassay data showed that the EGFP-tag did not change Cry11A toxicity against the homozygous AaeCad-EGFP mutants when compared with Orlando wild-type mosquitoes (S5 Fig).

**Fig 8:**
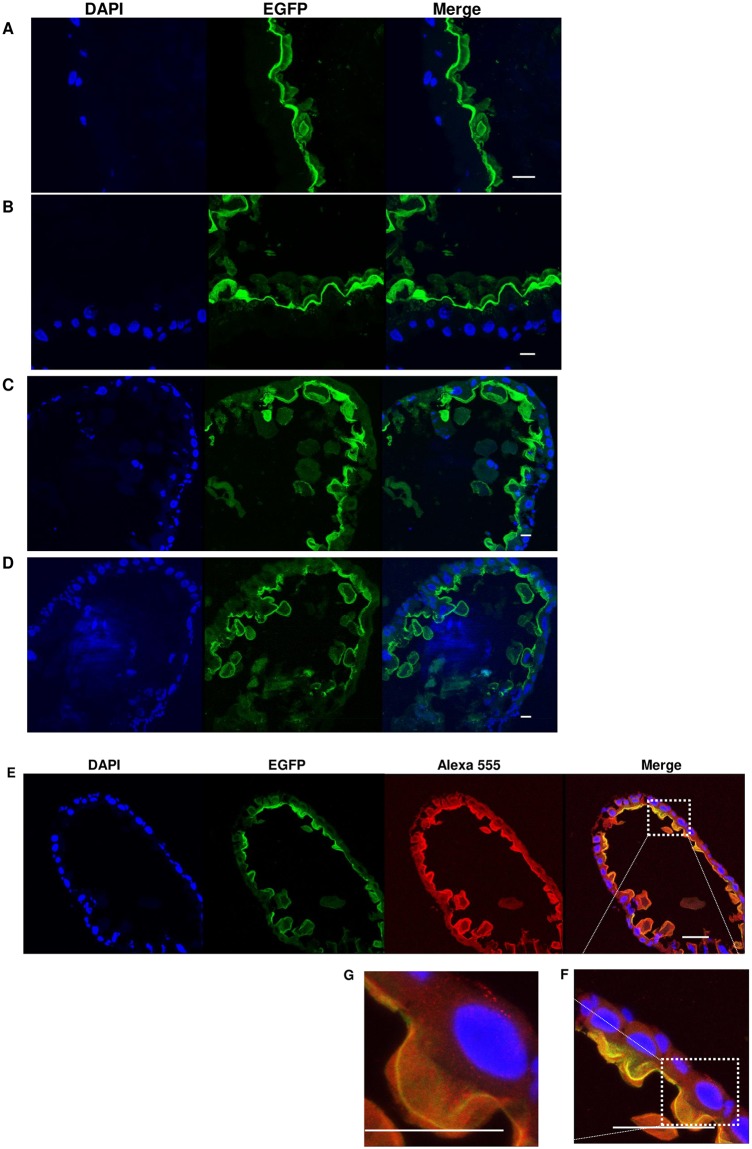
Larval exposure to low level Cry11A toxin concentrations disrupts mosquito midgut cells and causes these cells to shed its cell membrane. The larval midgut cell morphology was observed under a confocal microscope after the mosquitos were treated at the LC_10_ dose for 1 hr (A), 4 hr (B), 8 hr (C) and 16 hr (D). Changes in cell conformation were observed with Armadillo staining in cadherin-EGFP mosquitoes (E, F and G) at 18h. The same image is observed with DAPI, EFGP (cadherin) and Alexa 555 (Amardillo). Significant AaeCad-EGFP and Amardillo signaling is also observed in the intracellular compartments. Bar: 50μm.

The EGFP tagged AaeCad provides a better visualization of *Aedes* midgut cell structure. We therefore analyzed the effect of Cry11Aa toxin treatment on cell morphology in the posterior midgut. We monitored changes in cell morphology after 4^th^ instar mosquito larvae were treated with an LC_10_ dose of Cry11A toxin for 1h, 4h, 8h and 16h. In mosquito larvae that showed no change in feeding behavior or ability to move in the first hour, microvilli in the epithelial cells shrank and some of them were damaged (Fig 8A). At 4h, most microvilli had detached from the cells, but the epithelial cells remain intact (Fig 8B). At 8h, midgut epithelial cells were deformed, and some extracellular round-shaped membranes surrounded by clear green fluorescence membrane appeared in the lumen of mosquito guts (Fig 8C). Finally at 16h, more such membrane components were observed in the midgut lumen (Fig 8D). Apparently, in response to low levels of toxin the lumen-facing epithelial cell membrane is pinched off and lost from the epithelial cells forming extracellular round-shaped membranes. But the remaining part of the cell, which contains the nucleus, forms a new cell membrane to maintain cellular integrity (Fig 8E, 8F and 8G). Notably, Armadillo that co-localizes on the apical side of midgut epithelial cells with EGFP-tagged AaeCad, was also detected on the shed cell membranes and in the remaining cell membrane as is AaeCad-EGFP. Armadillo and EGFP-tagged AaeCad staining is also observed within the cell. Collectively, these images show that in both the cell membranes that are lost and that remaining in the cell, Armadillo always colocalizes with AaeCad-EGFP. It suggests a series of proteins are involved in the cell membrane shedding, probably including EGFP-tagged AaeCad and Armadillo proteins.

## Discussion

Our previous studies showed AaeCad is a functional receptor for the Cry11A toxin [28,29,41], and it also binds with high affinity a related mosquitocidal toxin, Cry11Ba [42]. AaeCad is homologous if not orthologous, to Cry toxin-binding cadherins identified in a number of the lepidopterans, including *Heliothis*, *Pectinophora*, *Helicoverpa*, *Bombyx* and *Manduca* [22–26], and also in coleopterans, *Tenebrio* and in *Leptinotarsa* [43,44]. However, all these “cadherin-like” receptor protein sequences do not align well with the established vertebrate cadherin family [45] or those from *Drosophila*; so they represent a novel and distinct cadherin family. Although their role in mediating Bt toxicity has been clearly documented, their physiological function remains unknown.

Hence with availability of ZFNs and then TALENs, we made knock outs of the *AaeCad* gene. While we obtained *Aedes* heterozygotes of these knockouts with both ZFN and TALEN (Figs 1 and 2) we were unable to obtain homozygotes, even though the ZFN and TALEN targets were in different exons. The knockouts made would have affected all the known *AaeCad* alleles. Therefore, we surmise from this data that this *Aedes* cadherin is likely essential, and that it differs from lepidopteran cadherins, even though they are at least homologous and they both bind Bt Cry toxins.

To analyze its role in *Aedes*, AaeCad-EGFP expression was monitored in larvae and adults. In both cases this cadherin was expressed exclusively in guts, and also in guts isolated from pupae, thus showing expression occurs in a tissue-specific manner (Figs 4 and 5). However, we cannot exclude minor levels of expression in other tissues and in the embryo.

Unless AaeCad is essential for the embryo development, we surmise that the lethality of *AaeCad* gene knockout might not be caused by the absence of AaeCad expression in other tissues but that in the midgut. Other than the cell-cell adhesion, cadherin proteins have also evolved on cell-cell recognition and sorting, cell and tissue polarity, boundary formation in tissues and coordination of multicell movement [46,47]. Apparently AaeCad is present only in the apical side of epithelial cells, so it is unlikely involved in the cell-cell adhesion. But it is possible AaeCad could play a role beyond that in cell-cell adhesion in the larval or adult midgut. In *Drosophila*, Cad99C, one of the 17 cadherin-like proteins in cadherin family localizes on the apical side of follicle cells and adult midgut cells, but it determines the microvillus length [48,49]. Therefore, this study provides a clue for further investigating the physiological function of “cadherin-like” receptor proteins for Bt Cry toxins.

Our prior data showed the Cry11A toxin binds to the apical side of epithelial cells in larval midguts and that an anti-AaeCad antibody also is localized in the same area [41]. In this study, we also observed that Cry11A bound to the apical side of larval gastric caecae (Fig 7A) and posterior midgut (Fig 7E) cells exactly where AaeCad-EGFP was expressed (Fig 6A and 6E). Thus, their co-localization further indicates that AaeCad is indeed a receptor for the Cry11A toxin. In addition, we detected weak AaeCad-EGFP expression in the anterior midgut, and this expression was not observed with anti-AaeCad staining, while expression in the gastric caeca and posterior gut was (Fig 2, [41]). Thus the low-level expression is more visible with gene tagging than by immunostaining.

With the homozygous AaeCad-EGFP mutant line we analyzed changes in *Aedes* midgut cell morphology after Cry11A toxin treatment for varying periods (Fig 8). At the LC_50_ levels, midgut cells were extensively damaged preventing us from obtaining any decent cell structures. However, using larvae that survived the LC_10_ dose of Cry11A toxin, we observed there were significant differences in the apical cell membrane structure. As expected, there was early loss of microvilli followed by the loss of cell membranes possibly by shedding, and the resulting thinner epithelium membrane (Fig 8). It appears the midgut cell response to toxin action is an attempt by the mosquito to maintain a functional midgut. Similarly, when *Drosophila* intestinal epithelial cells are exposed to the pore-forming toxin, hemolysin, secreted by *Serratia marcescens*, they undergo epithelium thinning followed by a recovery of epithelium thickness within a few hours[50]. Therefore, it is likely that epithelium thinning is a conserved mechanism for host defense against the pore-forming toxins.

In *Caenorhabditis elegans*, pores formed by Cry5B toxins are taken up from the plasma membrane into the cells by Rab5-controlled endocytosis, transported into lysosomes, and then expulsed into lumen by Rab11-controlled exocytosis [51]. Here we also observed some fluorescence inside midgut cells, but it is unknown if *Aedes* Rab5 and Rab11 homologs are involved in the cell’s defense against Cry11A toxin or other mosquitocidal toxins. Moreover, when the larvae were removed from additional toxin exposure, some of them developed normally (S2 Table). Since the Cry11A toxin forms pores [51], rapid loss of the cell membrane containing pores and receptors prevents further cell damage. Hence damaged cells can repair themselves by shedding their membrane that contains the toxin pores, unless toxin concentrations are high. Therefore, membrane shedding can be considered a defense mechanism.

Additionally, because the EGFP tagging does not alter Cry11A toxin binding and toxicity (Fig 7 and S5 Fig), the AaeCad-EGFP homozygous mutant line can be used to further interpret the molecular mechanisms of Cry11A toxicity. For example, the current model of Cry toxin interaction suggests the cadherin moves into lipid rafts after toxin binding to the cadherin and a GPI-anchored receptor [18,52]. Hence AaeCad-EGFP migration into lipid raft can be monitored by total internal reflection fluorescence microscopy. In addition, fluorescently tagged proteins in gene-modified insects can be used for tracing their release in the field. Finally, out study demonstrates that proteins in a non-model insect can be fluorescently-labeled in vivo, and where the homozygous mutant line of an essential gene can be generated successfully.

## Supporting information

S1 Fig Strategy for analyses of ZFN and TALEN mediated gene editing. ***A***. Analyses of ZFN and TALEN injected embryos and subsequent generations. ***B***. TALEN mRNA preparation.
(TIF)

S2 Fig CRISPR-Cas9 mediated tagging of the AaeCad. ***A***. Mosquito breeding diagram. ***B***. gRNA *in vitro* cleavage test. M, DNA Marker; Lane 1, plasmid pActin-Aaecad; Lane 2, mixture of plasmid pActin-Aaecad and gRNA; Lane 3, mixture of plasmid pActin-Aaecad and Cas9 protein; Lane 4, mixture of plasmid pActin-Aaecad, gRNA and protein Cas9. ***C***. gRNA *in vivo* cleavage test by high resolution melt (HRM) analysis. Seven out of twenty samples displayed differentiated HRM curves.
(TIF)

S3 Fig Three different *Aedes* Rad51 isoforms are observed in *Aedes* midgut. ***A***. Cloning of different *Aedes* Rad51 isoforms into the vector, pcDNA3.1. Lane M, DNA marker; Lane 1, pCR2.1-Rad51a digestion with *Nhe*I and *Xho*I restriction enzymes; Lane 2, pCR2.1-Rad51b digestion with *Nhe*I and *Xho*I restriction enzymes; Lane 3, pCR2.1-Rad51c pCR2.1-Rad51a digestion with *Nhe*I and *Xho*I restriction enzymes. ***B***. The Rad51c clone was used for mRNA preparation. Lane M, DNA Marker; Lane 1, untailed Rad51c mRNA; Lane 2, tailed Rad51 mRNA; Lane 3, purified tailed Rad51 mRNA.
(TIF)

S4 Fig Tissue localization of the AaeCad-EGFP protein during mosquito development. ***A***. Second instar larvae; B. Third instar larvae; C. Fourth instar larvae; D. Late forth instar larvae; E. pupae; F. male adult mosquito; G. female adult mosquito; H. AaeCad protein localization in adult female gut after blood feeding; The right two columns of images especially showed the fluorescence of AaeCad-EGFP in the cadia or gastric caecae. Bar: 500 μM.
(TIF)

S5 Fig Cry11A toxin is equally toxic to the EGFP-tagged Aaecad homozygous mosquitoes as to wild-type. Cry11A bioassay against the EGFP-tagged cadherin mutant (square) and wild-type (circle) mosquitoes. The data indicates EGFP-tagging did not change Cry11A toxicity to the homozygous cadherin-EGFP mutants.
(TIF)

S1 Table Bioinformatics analysis for G1 TALEN mosquitoes. (DOCX)

S2 Table LC_10_ dose of Cry11A toxicity on the *Aedes* mosquito larvae and adults. (DOCX)
