# Supplementary figures and images for: Aedes cadherin receptor that mediates Bacillus thuringiensis Cry11A toxicity is essential for mosquito development
Source: PLoS Negl Trop Dis. 2020 Feb 3;14(2):e0007948. doi: 10.1371/journal.pntd.0007948 (PMC7018227; doi:10.1371/journal.pntd.0007948)

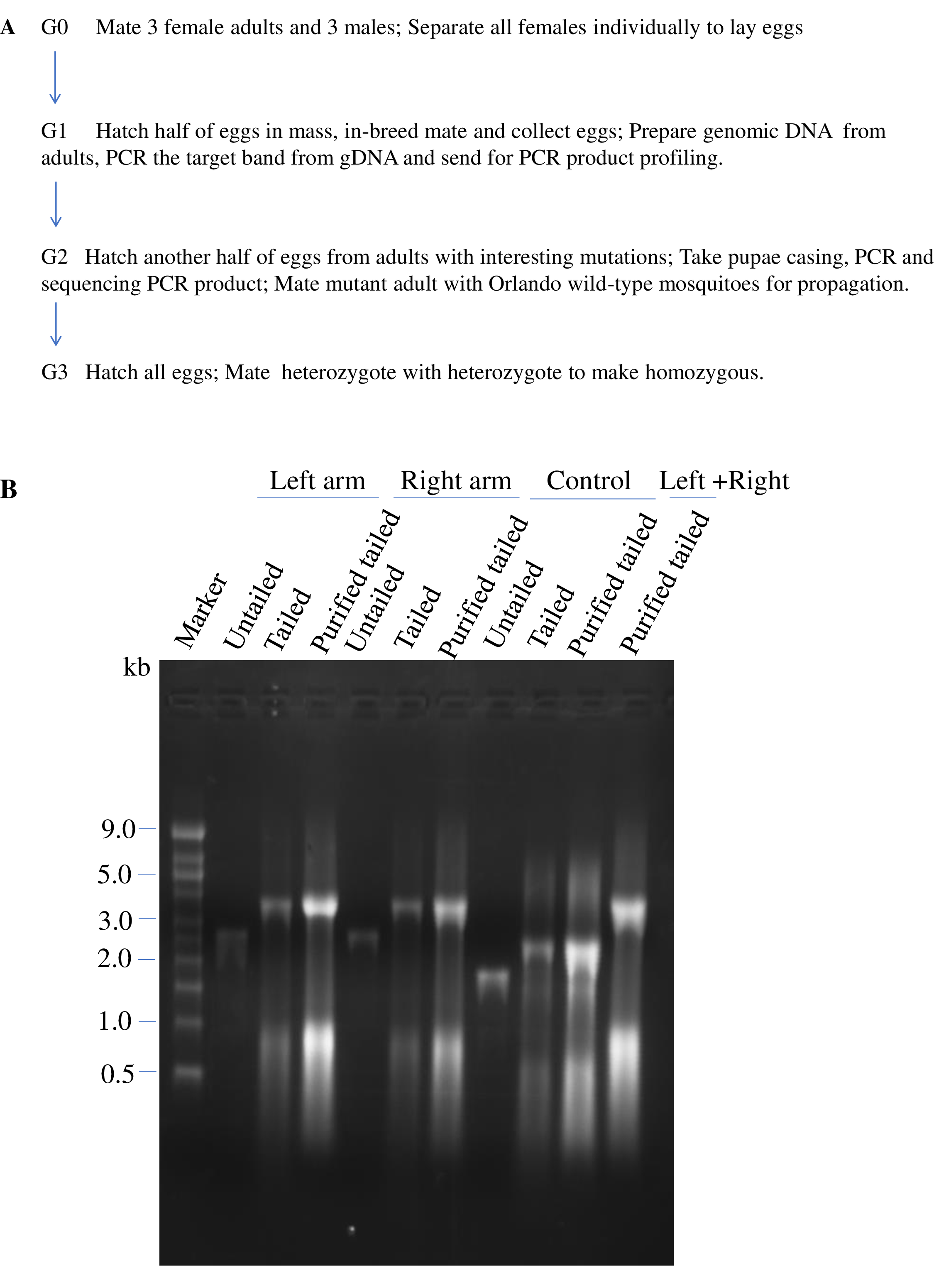

Supplement: S1 Fig — A. Analyses of ZFN and TALEN injected embryos and subsequent generations. B. TALEN mRNA preparation. (TIF) [file pntd.0007948.s001.tif]

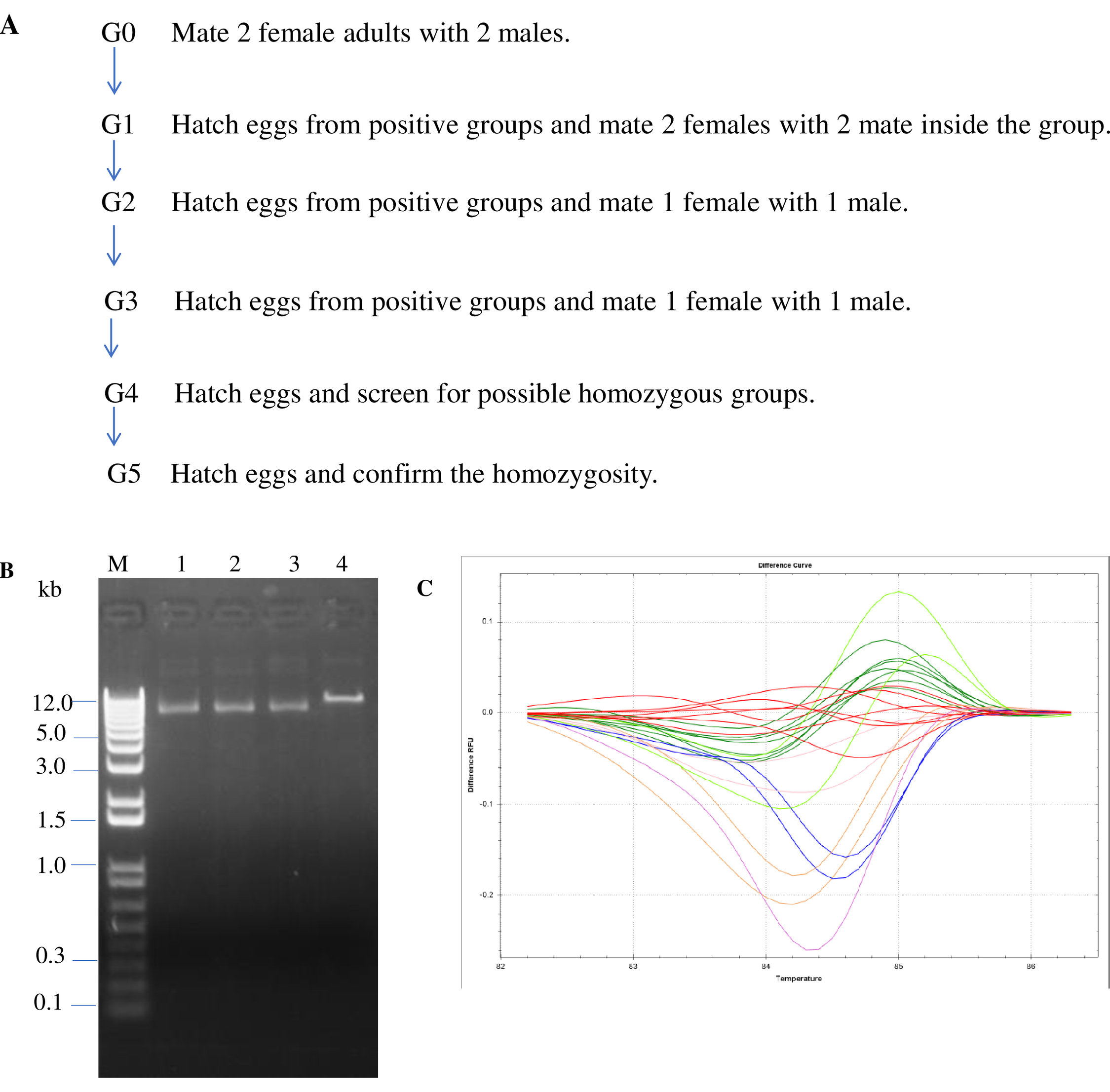

Supplement: S2 Fig — A. Mosquito breeding diagram. B. gRNA in vitro cleavage test. M, DNA Marker; Lane 1, plasmid pActin-Aaecad; Lane 2, mixture of plasmid pActin-Aaecad and gRNA; Lane 3, mixture of plasmid pActin-Aaecad and Cas9 protein; Lane 4, mixture of plasmid pActin-Aaecad, gRNA and protein Cas9. C. gRNA in vivo cleavage test by high resolution melt (HRM) analysis. Seven out of twenty samples displayed differentiated HRM curves. (TIF) [file pntd.0007948.s002.tif]

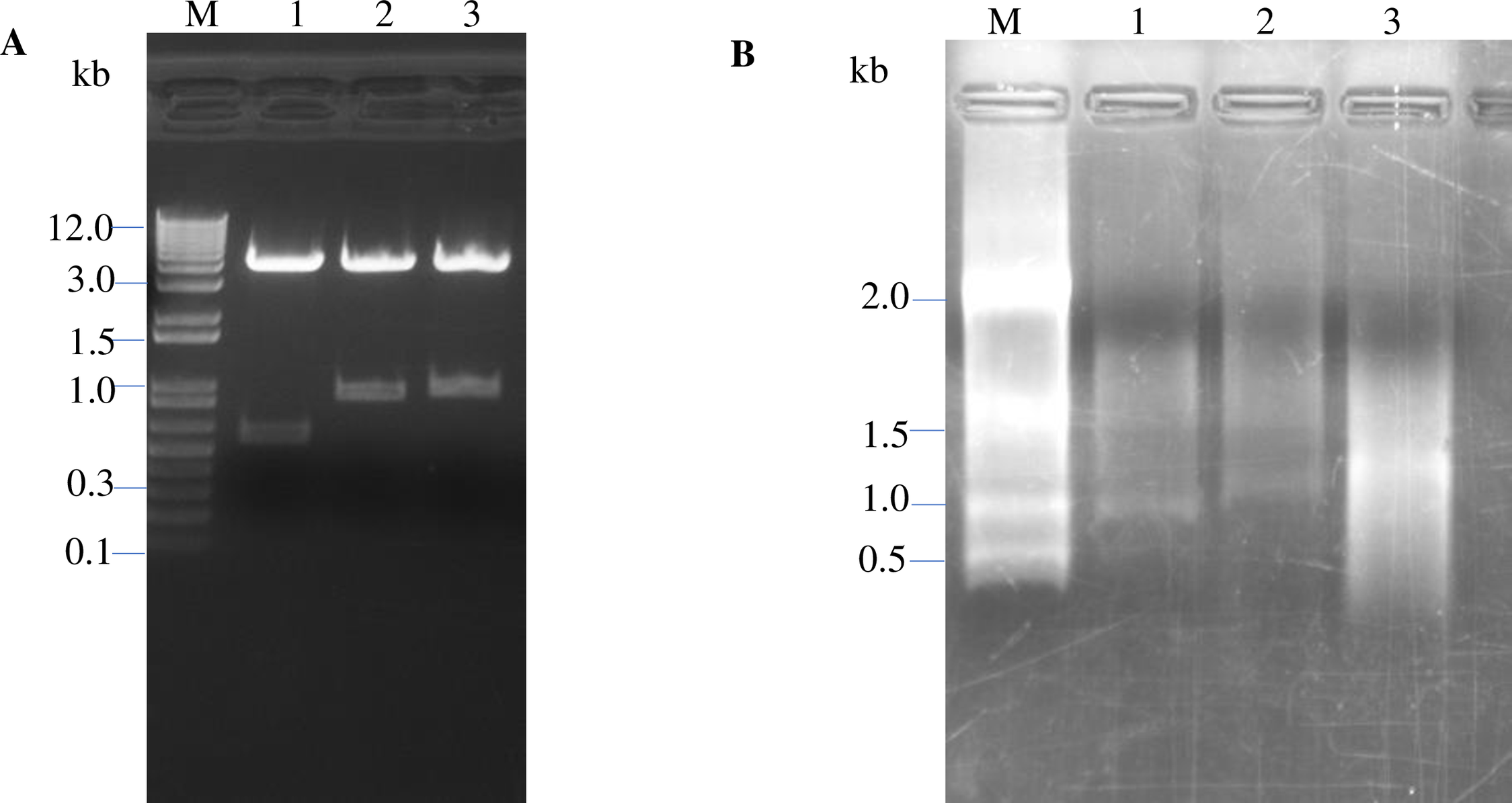

Supplement: S3 Fig — A. Cloning of different Aedes Rad51 isoforms into the vector, pcDNA3.1. Lane M, DNA marker; Lane 1, pCR2.1-Rad51a digestion with NheI and XhoI restriction enzymes; Lane 2, pCR2.1-Rad51b digestion with NheI and XhoI restriction enzymes; Lane 3, pCR2.1-Rad51c pCR2.1-Rad51a digestion with NheI and XhoI restriction enzymes. B. The Rad51c clone was used for mRNA preparation. Lane M, DNA Marker; Lane 1, untailed Rad51c mRNA; Lane 2, tailed Rad51 mRNA; Lane 3, purified tailed Rad51 mRNA. (TIF) [file pntd.0007948.s003.tif]

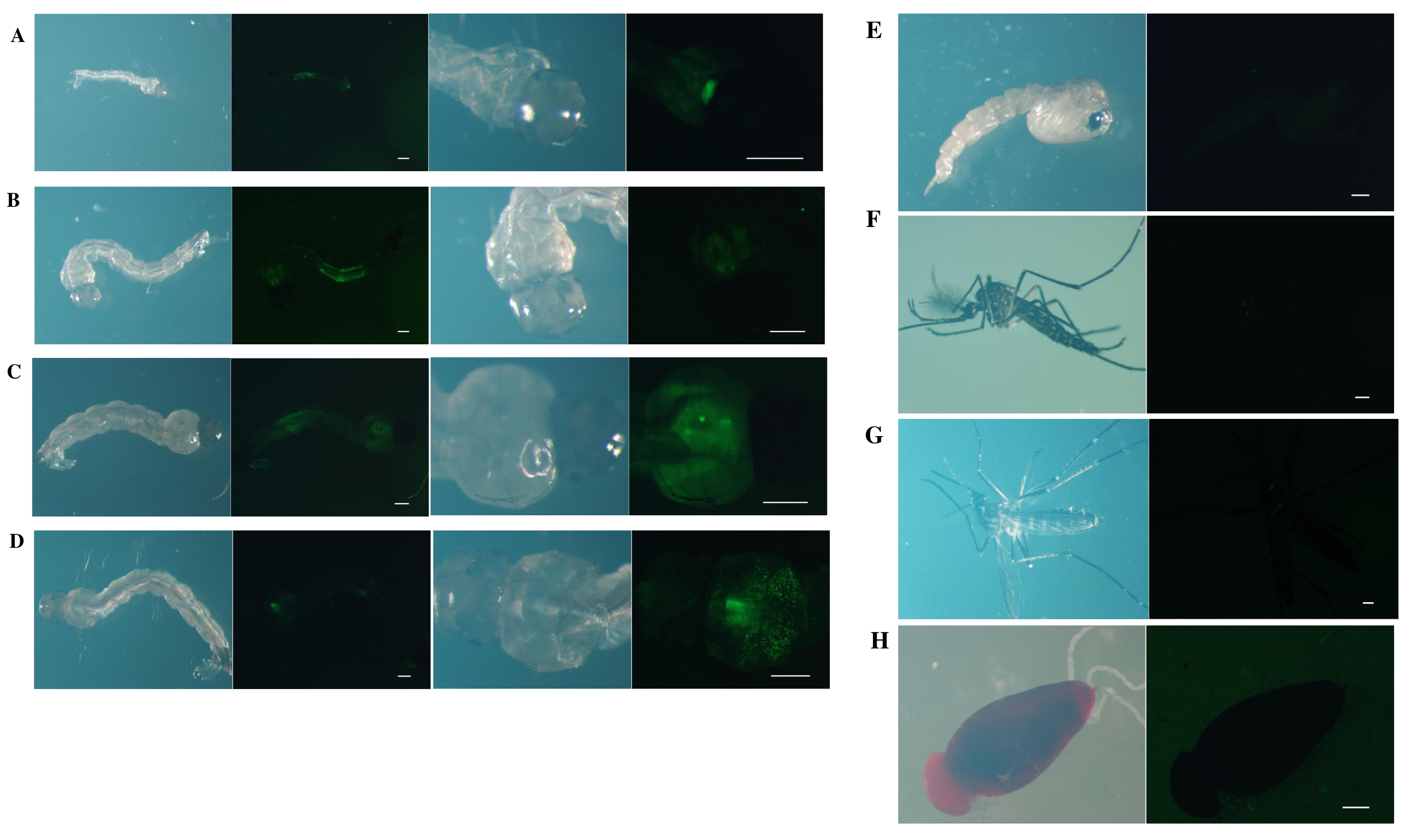

Supplement: S4 Fig — A. Second instar larvae; B. Third instar larvae; C. Fourth instar larvae; D. Late forth instar larvae; E. pupae; F. male adult mosquito; G. female adult mosquito; H. AaeCad protein localization in adult female gut after blood feeding; The right two columns of images especially showed the fluorescence of AaeCad-EGFP in the cadia or gastric caecae. Bar: 500 μM. (TIF) [file pntd.0007948.s004.tif]

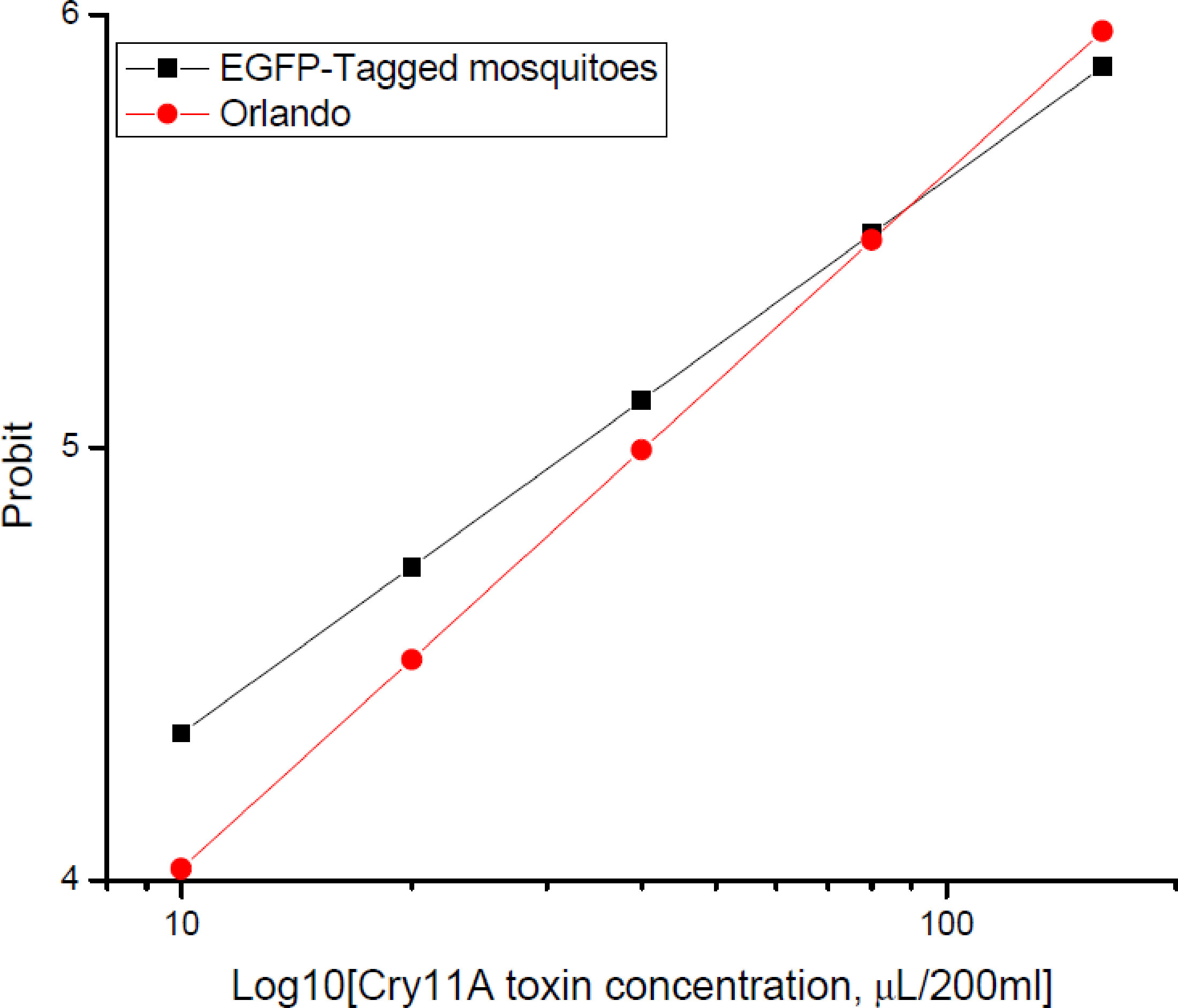

Supplement: S5 Fig — Cry11A bioassay against the EGFP-tagged cadherin mutant (square) and wild-type (circle) mosquitoes. The data indicates EGFP-tagging did not change Cry11A toxicity to the homozygous cadherin-EGFP mutants. (TIF) [file pntd.0007948.s005.tif]
