# Supplementary material for: Aedes cadherin receptor that mediates Bacillus thuringiensis Cry11A toxicity is essential for mosquito development
Source: PLoS Negl Trop Dis. 2020 Feb 3;14(2):e0007948. doi: 10.1371/journal.pntd.0007948 (PMC7018227; doi:10.1371/journal.pntd.0007948)
Supplement: S1 Table — (DOCX) [file pntd.0007948.s006.docx]

**S1 Table Bioinformatics analysis for G1 TALEN mosquitoes**

| Mosquito | Indels | Total reads | Altered reads | Percentage |
| --- | --- | --- | --- | --- |
| 31M | 4 | 6664 | 1169 | 17.5 |
| 32M | 4 | 11184 | 1107 | 9.89 |
|  | 3 | 11184 | 3314 | 29.6 |
| 41 | 3 | 18190 | 1383 | 7.6 |
| 70F | 6 | 45241 | 2745 | 6.07 |
