# Supplementary material for: Aedes cadherin receptor that mediates Bacillus thuringiensis Cry11A toxicity is essential for mosquito development
Source: PLoS Negl Trop Dis. 2020 Feb 3;14(2):e0007948. doi: 10.1371/journal.pntd.0007948 (PMC7018227; doi:10.1371/journal.pntd.0007948)
Supplement: S2 Table — (DOCX) [file pntd.0007948.s007.docx]

**S2 Table LC_10_ dose of Cry11A toxicity on the *Aedes* mosquito larvae and adults**

| **Group** | **Dose (ng/ml)** | **Larvae** | **Mortality in 1 day (%)** | **Mortality in 3 days (%)** | **Adult rate (%)** |
| --- | --- | --- | --- | --- | --- |
| 9-8 | 0 | 40 | 0 | 0 | 100 |
|  | 100 | 40 | 10 | 55 | 45 |
| Orlando | 0 | 40 | 0 | 0 | 100 |
|  | 100 | 40 | 12.5 | 55 | 47.5 |
